# Misdiagnosis of occult heterotopic intramural ectopic pregnancy by two-dimensional ultrasound: Two case reports

**DOI:** 10.3389/fmed.2025.1620400

**Published:** 2025-10-01

**Authors:** Jian Zhang, Zeyang Dong, Shaofeng Guo, Hongxia Ma, Bin Huang

**Affiliations:** ^1^Department of Ultrasound, Zhejiang Hospital, Hangzhou, Zhejiang, China; ^2^The Second School of Clinical Medicine, Zhejiang Chinese Medical University, Hangzhou, Zhejiang, China

**Keywords:** case description, ultrasound, transvaginal sonography, ectopic pregnancy, MRI

## Abstract

We report two cases of heterotopic intramural ectopic pregnancy (HIMP) following multiple abortions, with lesions located near the uterine fundus or uterine horn. These cases are particularly challenging to diagnose using early two-dimensional transvaginal sonography (TVS). We analyzed multimodal imaging in combination with transabdominal ultrasound (TAS), color Doppler flow imaging (CDFI), three-dimensional TVS, and magnetic resonance imaging (MRI) for early diagnosis and ultimately removed the gestational sac tissue via hysteroscopy. The combination of multimodal imaging techniques, serum *β*-human chorionic gonadotropin (β-hCG) levels, and clinical vigilance should be considered to prevent the catastrophic consequences of HIMP, particularly when conventional two-dimensional TVS results are equivocal or misleading.

## Introduction

Intramural pregnancy (IMP) occurs when a pregnancy implants within the uterine wall, surrounded by the myometrium, and is not connected to the uterine cavity or fallopian tubes. IMP is an extremely uncommon type of ectopic pregnancy (EP), accounting for less than 1% of all ectopic pregnancies. Heterotopic pregnancy (HP) is the simultaneous occurrence of intrauterine pregnancy and ectopic pregnancy, while heterotopic intramural ectopic pregnancy (HIMP) is even rarer, with fewer than 50 documented cases in the literature (1, 2). The exact pathogenesis of IMP is unknown; however, uterine trauma, pelvic surgery, and *in vitro* fertilization (IVF) have been identified as potential risk factors in documented cases, with a history of curettage being the most commonly reported risk factor (3–5).

The clinical urgency of IMP arises from its propensity to cause catastrophic uterine rupture and hemorrhage. As the gestational sac implants into the myometrium, its expansion progressively erodes myometrial integrity, ultimately leading to life-threatening hemorrhage. Once detected, prompt and aggressive treatment is required, typically through hysterectomy, minimally invasive laparoscopic surgery, or medical management (4). Therefore, early diagnosis of IMP is crucial (6). According to the European Society of Human Reproduction and Embryology (ESHRE) ultrasound classification criteria, IMP can be divided into complete and incomplete types, each requiring different therapeutic approaches (7). When diagnosed early, IMP can be managed with conservative therapy or minimally invasive surgery, which may preserve the patient’s reproductive function (8). However, in the early stages of the disease, differentiating IMP from other diagnoses, such as intrauterine pregnancy, residual abortion, or trophoblastic diseases, can be challenging, particularly in cases of interstitial pregnancy (ITP), due to their clinical and ultrasound similarities (9). Distinguishing IMP from ITP can be especially difficult during the initial two-dimensional transvaginal sonography (TVS) examination. Repeating the ultrasound, along with three-dimensional TVS, magnetic resonance imaging (MRI), or even laparoscopy, may provide significant diagnostic assistance (10, 11).

## Case presentation

### Case 1

The patient, a 30-year-old woman, presented to the hospital after 2 months of amenorrhea with a positive urine pregnancy test. She had previously undergone two hysteroscopic abortions (G3P0). Serum *β*-human chorionic gonadotropin (β-hCG) levels were measured at over 5,000 mIU/mL (normal <5 mIU/mL). Two-dimensional TVS showed three dark area-like echoes at the base of the uterus, with a yolk sac and germinal structures visible in the largest dark zone, and the length of the embryo was approximately 3.9 mm. Since the patient had no desire to have children, we performed a hysteroscopic suction curettage. Clear chorionic tissue was found on pathologic examination of the specimen.

**Figure 1:**
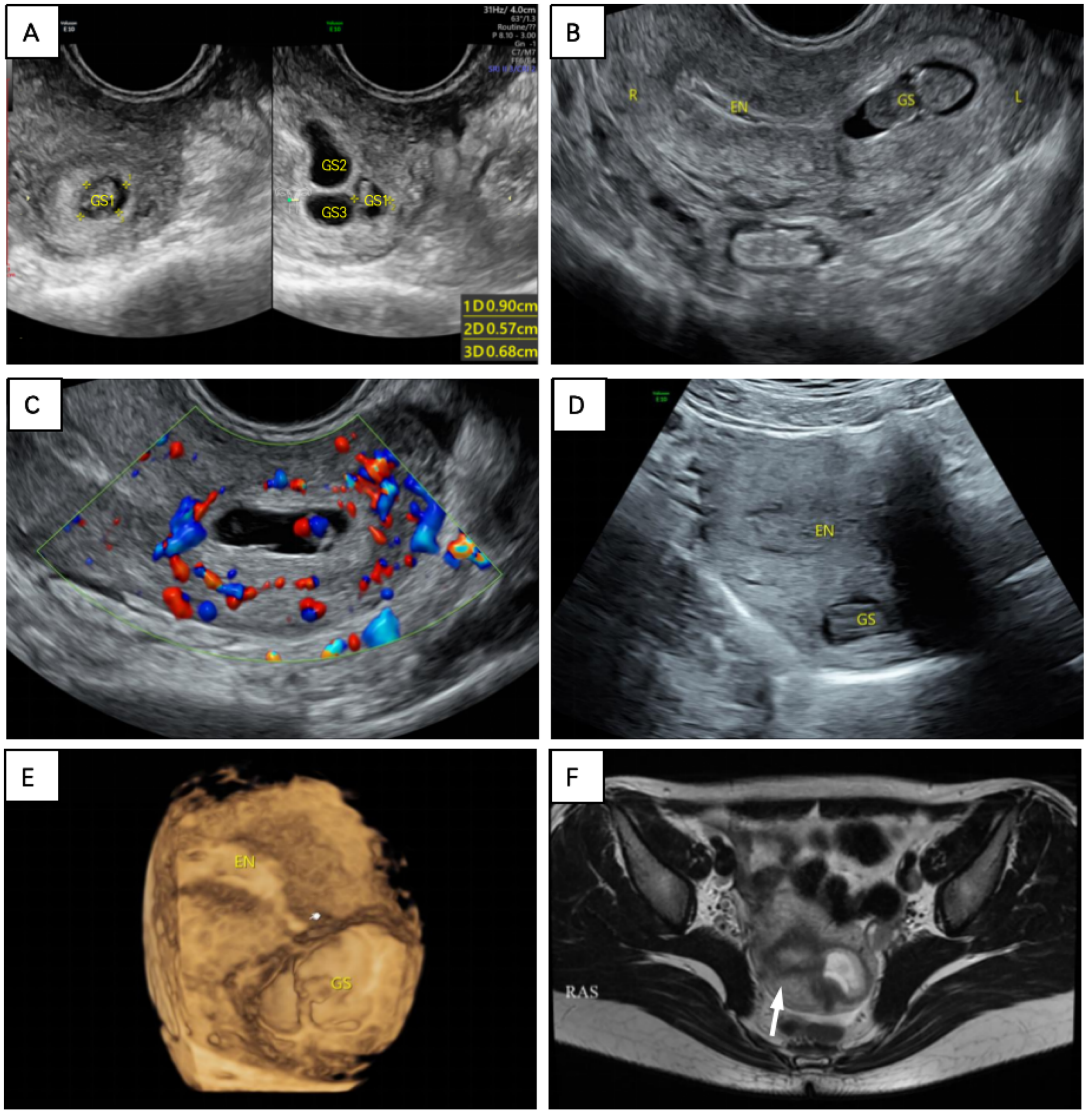
**(A)** Initial transvaginal ultrasound revealed three hypoechoic areas, one with internally visible yolk sacs and embryonic buds. **(B)** The gestational sac at the base of the uterus was observed again on transvaginal ultrasound 1 week after curettage. **(C)** CDFI appeared to detect myometrial blood flow between the sac and the endometrium. **(D)** Transabdominal ultrasound and **(E)** three-dimensional ultrasound imaging revealed that the sac was outside the endometrium (The white finger indicates the endometrial end of the uterus). **(F)** On the T2-weighted MRI sequence, where the white arrow points to, the high signal area is the endometrium, the surrounding low signal area is the union zone, and the left high-signal gestational sac is located outside the endometrium.

One week postoperatively, serum *β*-hCG levels unexpectedly rose to 14,676 mIU/mL. To rule out a trophoblastic tumor, we performed another vaginal ultrasound, which revealed an enlarged gestational sac at the uterine fundus, accompanied by fetal cardiac activity. We also observed a lack of continuity between the gestational sac and the endometrium. Color Doppler flow imaging (CDFI) revealed abundant blood flow signals between the gestational sac and the endometrium, which closely resembled myometrial blood flow signals. Three-dimensional TVS revealed that the uterine fundus protruded outward locally. MRI indicated the presence of the uterine junction zone and confirmed that the gestational sac was located within the myometrium, rather than in the endometrium (Figure 1).

**Figure 2:**
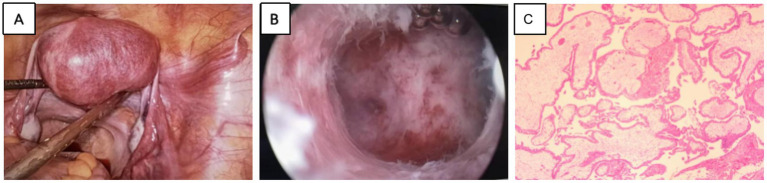
**(A)** Laparoscopic visualization of localized outward expansion of the left myometrium. **(B)** Visualization of an internal gestational sac in the myometrium after myometrial incision under hysteroscopy. **(C)** Postoperative pathological confirmation of IMP.

Considering the patient’s history, we considered the diagnosis to be an intrauterine pregnancy complicated by a fundic intramural pregnancy. We then performed a laparoscopy and identified a slightly elevated, vascularized mass on the left anterior aspect of the uterus. Given the severity of the condition and the extremely high serum *β*-hCG levels, we proceeded with hysteroscopy under laparoscopic surveillance after obtaining the patient’s informed consent. The endometrium and myometrium of the left posterior uterine wall were successfully electrocoagulated and incised, allowing for the removal of the gestational sac. Postoperative pathological examination confirmed the presence of chorionic tissue (Figure 2). By postoperative day 4, serum *β*-hCG levels had decreased to 1,672 mIU/L and returned to normal within 3 weeks without any complications.

### Case 2

The patient, a 41-year-old woman, presented with intermittent vaginal bleeding lasting more than 2 weeks. Her medical history revealed that she had undergone hysteroscopic abortions over the past 2 years (G3P1). Two months ago, she underwent a medical abortion with mifepristone and misoprostol, which successfully expelled the gestational sac-like tissue. Following this procedure, her serum *β*-hCG levels decreased from 67,055 to 2,469 mIU/mL.

**Figure 3:**
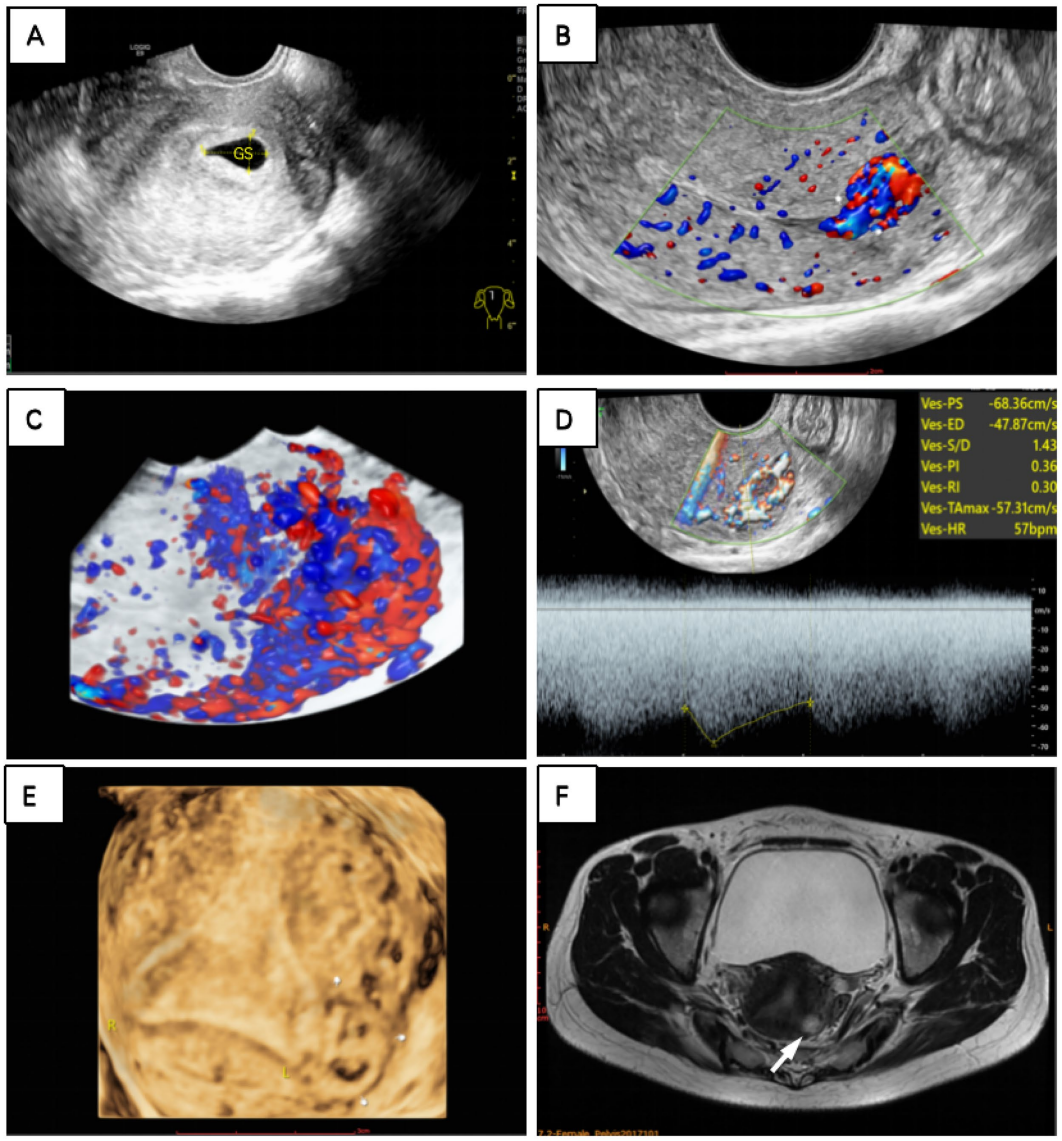
**(A)** A hypoechoic area was observed in the uterus on transvaginal ultrasound. Two months after medication abortion, **(B)** CDFI and **(C)** three-dimensional flow imaging revealed abundant blood flow signals resembling vascular pools at the uterine fundus. **(D)** Spectral Doppler revealed high-speed, low-resistance arteriovenous fistula-like flow. **(E)** Three-dimensional ultrasonography and **(F)** MRI T2-weighted sequence revealed a gestational sac within the left myometrium, outside the endometrial cavity (white finger and arrow pointing to the gestational sac).

However, a 2-month follow-up revealed a paradoxical elevation in serum β-hCG levels to 3,069 mIU/mL, prompting further diagnostic evaluation. Initially, we suspected that an incomplete medical abortion with retained tissue was the cause, so we performed a two-dimensional TVS. However, it did not reveal any obvious gestational sac or mass in the uterine cavity, but abundant blood flow signals extending from the uterine cavity to the muscularis propria were detected on the CDFI mode. Three-dimensional TVS revealed an abnormal echogenic mass at the fundus of the uterus, which appeared on color Doppler flow imaging as a garland of abundant and disorganized flow signals extending into the muscularis propria. The mass showed a low-resistance, high-velocity blood flow spectrum, with a resistance index of 0.30, a pulsatility index of 0.36, and a peak systolic velocity of 68.36 cm/s (Figure 3).

An incomplete intramural pregnancy was diagnosed, and the gestational sac was removed hysteroscopically under laparoscopic surveillance. Postoperative pathology of the intramural pregnancy showed abundant chorionic tissue and trophoblast cells, confirming the diagnosis. One week after surgery, the follow-up showed that serum *β*-hCG levels had decreased to 162.90 mIU/mL.

## Discussion

Our case series underscores the limitations of conventional imaging modalities. In case 1, two-dimensional TVS initially misdiagnosed the condition as an intrauterine pregnancy combined with partial gravidity or degeneration of uterine fibroids, and the location of the gestational sac was mistakenly assumed to be intraluminal due to overlapping gestational sacs and myxoid edema. The misjudgment of the location of the gestational sac resulted in the patient needing an additional hysteroscopic clearance procedure and delayed diagnosis of IMP. In case 2, probably due to the small size of the early gestational sac, two-dimensional TVS failed to detect the IMP within the myometrium and instead diagnosed an early intrauterine pregnancy. This resulted in the patient undergoing an additional medication abortion before laparoscopy and hysteroscopic resection were performed. Fortunately, no serious complications occurred in any of these cases, thanks to accurate diagnosis by three-dimensional TVS and MRI.

Retrospective image analysis revealed two distinctive features in our cohort compared to existing literature. First, there was an unusual proximity between the gestational sacs and the endometrium. According to the ESHRE ultrasound classification criteria, we consider this to be an incomplete IMP, but it may also be associated with a combined intrauterine pregnancy (7, 12, 13). Second, on close inspection using CDFI, an abnormally high blood flow signal was observed between the gestational sac and the endometrium, resembling the myometrium. This was characterized by high velocity and low resistance on spectral Doppler, with arteriovenous shunting phenomena noted (9, 13).

In clinical practice, differentiating between IMP, interstitial ectopic pregnancy, and subserosal ectopic pregnancy (SP) is crucial, as they require different treatment approaches. SP is a rare variant of IMP, where the gestational sac is primarily enclosed by the uterine serosal layer and requires surgical intervention (14). The four diagnostic criteria for SP have been well summarized by Stabile et al. (15): an empty uterine cavity, normal fallopian tubes and ovaries, myometrial invasion <50% from the external uterine wall, and a history of previous uterine muscle layer surgery. Interstitial pregnancy occurs in the interstitial portion of the fallopian tube, where the gestational sac is connected to the fallopian tube rather than the uterine cavity. The outer muscle layer of the gestational sac in interstitial pregnancy is typically thin (<5 mm), and the ultrasound may reveal an interstitial line sign. Interstitial pregnancy is located lateral to the round ligament, whereas IMP occurs within the myometrial layer, independent of the round ligament’s position (16, 17).

Multimodal imaging modalities have proven to be crucial for accurate diagnosis. Three-dimensional TVS and transabdominal ultrasound (TAS) provide improved spatial resolution, while magnetic resonance imaging (MRI) allows for more comprehensive visualization of the anatomical location of the gestational sac, as well as the growth of the myometrium and the depth of IMP infiltration within the myometrium (18, 19).

The treatment of IMP is tailored according to the type of pregnancy and serum *β*-hCG levels (20). In incomplete IMP, if serum β-hCG levels are low and the pregnancy is detected early, medical management may be considered. However, if the pregnancy progresses, surgical intervention is usually required, and laparoscopic-assisted hysteroscopic resection is commonly used to preserve fertility. In complete IMP, surgery is generally necessary, and a hysterectomy may be performed if there is a risk of rupture or severe complications. For milder cases, laparoscopic or hysteroscopic resection may be performed. Serum *β*-hCG levels play a crucial role in treatment decisions; higher levels (typically >10,000 mIU/mL) usually indicate the need for surgery, while lower levels may allow for conservative management or monitoring (21).

These two patients had a history of previous abortion procedures without uterine plasma layer defects or endometriosis, so the main consideration was the occurrence of IMP in combination with intrauterine pregnancy, likely due to endometrial damage caused by multiple abortion procedures. This emphasizes the need for clinical vigilance in at-risk populations, especially those who have undergone uterine surgery, have adenomyosis, or have been exposed to assisted reproductive technologies. These patients require systematic evaluation of the myometrium using multimodal imaging modalities, along with rigorous postoperative serum *β*-hCG monitoring after abortion. If serum β-hCG levels persistently or atypically increase, suspicion of HIMP should be immediately raised, prompting multimodal assessment. This diagnostic paradigm emphasizes the critical role of combining multimodal imaging techniques with clinical vigilance to prevent catastrophic outcomes, especially when conventional two-dimensional TVS findings are equivocal or misleading.

## Data Availability

The original contributions presented in the study are included in the article/supplementary material, further inquiries can be directed to the corresponding author.
